# Obesity Paradox in Takotsubo Syndrome Among Septic ICU Patients: A Retrospective Cohort Study

**DOI:** 10.3390/jcm14082635

**Published:** 2025-04-11

**Authors:** Shreyas Yakkali, Raksheeth Agarwal, Aman Goyal, Yutika Dongre, Ankit Kushwaha, Ankita Krishnan, Anika Sasidharan Nair, Balaram Krishna Jagannayakulu Hanumantu, Aanchal Gupta, Leonidas Palaiodimos, Perminder Gulani

**Affiliations:** 1 Department of Medicine, Jacobi Medical Center/Albert Einstein College of Medicine, Bronx, NY 10461, USA; agarwalr1@nychhc.org (R.A.); dongrey@nychhc.org (Y.D.); ak1887@gmail.com (A.K.); leonidas.palaiodimos@nychhc.org (L.P.); perminder.gulani@nychhc.org (P.G.); 2 Seth GS Medical College and KEM Hospital, Mumbai 400012, India; amanmgy@gmail.com; 3 Department of Medicine, Critical Care Division, Cleveland Clinic, Cleveland, OH 44195, USA; 4 Department of Cardiovascular Medicine, University of Pennsylvania, Philadelphia, PA 19104, USA; h.balaram.krishna@outlook.com; 5 Department of Medicine, Beth Israel Lahey Health, Burlington, MA 01805, USA; draanchalgupta@gmail.com

**Keywords:** Takotsubo Syndrome (TTS), sepsis, obesity paradox, intensive care unit (ICU), shock index, body mass index (BMI), congestive heart failure (CHF)

## Abstract

**Background:** Takotsubo Syndrome (TTS) is a transient left ventricular systolic dysfunction typically characterized by anteroseptal-apical dyskinetic ballooning of the left ventricle with a hyperkinetic base, without significant obstructive coronary artery disease. The interplay between systemic inflammation and hemodynamic stress in sepsis exacerbates susceptibility to TTS. We aim to investigate the characteristics and factors associated with TTS in critically ill patients with sepsis admitted to the intensive care unit. **Methods:** A retrospective cohort study was conducted on 361 patients admitted to the medical ICU at a tertiary care hospital in New York City. All patients underwent transthoracic echocardiography (TTE) within 72 h of sepsis diagnosis. Patients were divided into TTS and non-TTS groups. Clinical data, comorbidities, and hemodynamic parameters were extracted from electronic medical records and analysed using multivariate logistic regression to determine independent predictors of TTS. **Results:** Among 361 patients, 24 (6.65%) were diagnosed with TTS. Female sex (OR 3.145, 95% CI 1.099–9.003, *p* = 0.033) and higher shock index (OR 4.454, 95% CI 1.426–13.910, *p* = 0.010) were significant predictors of TTS. Individuals with ≥ 25 kg/m^2^ had a lower odds of developing TTS as compared to their obese counterparts (OR 0.889, 95% CI 0.815–0.969, *p* = 0.007). **Conclusions:** The findings highlight that Female sex, higher shock index and a BMI < 25 kg/m^2^ emerge as possible predictors for development of TTS in patients with sepsis. Further research is needed to unravel the mechanisms behind the “obesity paradox” in TTS and optimize clinical strategies for high-risk patients.

## 1. Introduction

Sepsis is characterized by a dysregulated systemic inflammatory response to an infectious pathogen [1]. Despite advancements in understanding the immune pathways involved in this host response, sepsis remains a complex and challenging syndrome frequently associated with poor outcomes [2]. Annually, the Centres for Disease Control and Prevention reported that a minimum of 1.7 million adults in the U.S. are diagnosed with sepsis, resulting in at least 350,000 deaths [3].

Takotsubo Syndrome (TTS) is defined by a transient left ventricular systolic dysfunction typically characterized by anteroseptal-apical dyskinetic ballooning of the left ventricle with a hyperkinetic base, without significant obstructive coronary artery disease (CAD), with spontaneous resolution to normal left ventricular function over time [4]. Patients with TTS may present with a range of clinical symptoms, and the syndrome is frequently triggered by significant emotional stress or severe physical illness, which activates the sympathetic nervous system [5,6]. Over the past few decades, our understanding of TTS has grown, leading to the prompt use of coronary angiography in patients with acute cardiac chest pain to exclude obstructive coronary disease. TTS can be rapidly diagnosed owing to advancements in modern cardiac imaging [5]. Male sex, left ventricular ejection fraction (LVEF) < 45%, and concurrent acute neurological events predict failure of early recovery (within 10 days) of regional wall motion abnormalities (RWMAs) in TTS, which is associated with poorer long-term survival. Other predictors of poor long-term survival include older age, physical stress, non-typical ballooning patterns, and presence of late Gadolinium enhancement on cardiac magnetic resonance imaging [7]. Additionally, some patients with TTS may develop left ventricular outflow tract obstruction phenotype, which is associated with rapid hemodynamic compromise and an increased risk of cardiogenic shock and in-hospital mortality [8].

Obesity is a well-known cardiovascular risk factor. The term ‘obesity paradox’ refers to the counterintuitive observation where obese individuals have paradoxically better outcomes in cardiovascular conditions (e.g., coronary artery disease, heart failure, atrial fibrillation) compared to normal weight or underweight individuals. The underlying mechanisms of obesity paradox are not fully understood but may include factors such as higher metabolic reserves and differences in biomarkers and inflammatory responses [9].

The association between TTS development and sepsis is complex, and several mechanisms have been proposed. First, sepsis-induced systemic inflammation, along with inflammatory mediators and microbial byproducts, may contribute to myocardial dysfunction. Second, catecholamine toxicity affects cardiac function through sympathetic nervous system (SNS) activation. Third, exogenous administration of catecholamines occurs in individuals with septic shock. Finally, myocardial demand ischemia can result from insufficient coronary blood supply during infection [6]. The exact demographic heterogeneity, prevalence, risk factors, and outcomes of patients who develop TTS after initial admission for sepsis remain relatively understudied and unknown. Recent studies have explored the role of obesity in the development of TTS in patients with sepsis; however, the data are conflicting [10,11]. Our study aimed to address this gap in the literature by providing real-world evidence from a single institution in New York, USA.

## 2. Methods

In this retrospective study, we reviewed medical records of all patients admitted to the 12-bed medical intensive care unit (ICU) of a municipal hospital in New York with a diagnosis of sepsis between 1 January 2016, and 31 December 2017. Sepsis was identified based on the Third International Consensus Definitions for Sepsis and Septic Shock, characterized by life-threatening organ dysfunction resulting from a dysregulated host response to infection, indicated by an increase of at least 2 points in the Sequential Organ Failure Assessment (SOFA) score [1]. Infection was defined by the detection of microorganisms in culture or by radiologic and clinical findings consistent with infection, even in cases where cultures were negative. This study received approval from the Institutional Review Board of Albert Einstein College of Medicine (IRB No. 2018-8773). The requirement for informed consent was waived due to the study’s retrospective design. All authors affirm the accuracy and completeness of the data and the adherence of the final study to the approved research protocol.

**Figure 1:**
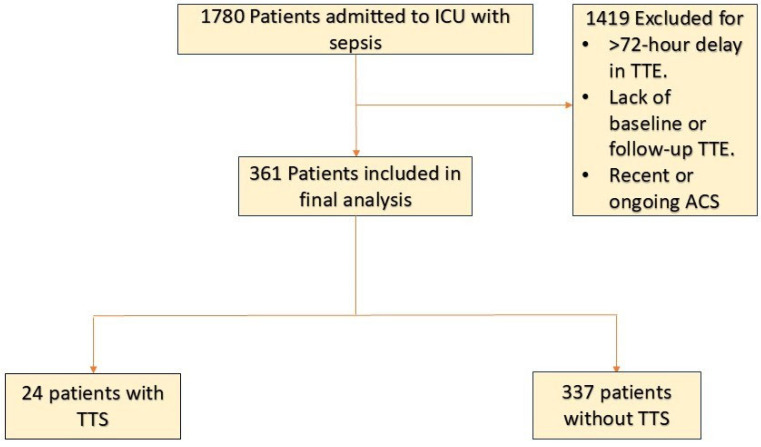
**Patient-Flowchart** Abbreviations: ACS, acute coronary syndrome; ICU, intensive care unit; LVEF, left ventricular ejection fraction; TTE, transthoracic echocardiography; TTS, takotsubo syndrome.

Medical records were reviewed for all adult patients (aged ≥ 18 years) admitted to the medical ICU with an initial diagnosis of sepsis determined by the admitting team. Patients were included in the study if they underwent a transthoracic echocardiogram (TTE) within 72 h of admission and had a comparison TTE conducted either within six months prior to admission (baseline TTE) or within three months following the diagnosis of sepsis (follow-up TTE). Patients were divided into TTS and non-TTS groups. The TTS group included patients with echocardiographic findings consistent with: transient akinesia or dyskinesia of the left ventricular mid-segments with or without apical involvement in the absence of significant CAD [12,13,14]. Patients were excluded if they met any of the following criteria: a recent history of acute coronary syndrome or heart failure exacerbation, or a primary diagnosis of acute coronary syndrome during the index admission Figure 1.

Patient demographics and baseline characteristics collected included age, sex, body mass index, smoking history, race, and ethnicity. Medical history was recorded, including hypertension, diabetes mellitus (DM), prior symptomatic heart failure, angiographically confirmed coronary artery disease, cirrhosis, atrial fibrillation, human immunodeficiency virus infection, prior cerebrovascular disease, and history of active or prior cocaine and/or ethanol use. Medication use was noted, including beta-blockers, angiotensin-converting enzyme inhibitors, angiotensin receptor blockers, mineralocorticoid receptor antagonists, and anticoagulants. Clinical characteristics, including Glasgow Coma Scale at sepsis diagnosis, blood pressure, heart rate, respiratory rate, temperature, and the need for vasopressors, mechanical ventilation, and/or renal replacement therapy, were also documented. Laboratory characteristics collected included complete blood count, electrolytes, renal and hepatic function, arterial blood gases, coagulation profile, troponin T, lactate levels, and microbiological data. Echocardiographic data regarding left and right ventricular (LV and RV) function and size were also collected. Culture positivity was defined by the growth of at least one microorganism from body fluids (e.g., urine, blood, sputum, bronchoalveolar lavage, cerebrospinal fluid, or peritoneal fluid) and/or biopsy specimens. These variables were used to calculate the Acute Physiology and Chronic Health Evaluation (APACHE) II score at admission and the Sequential Organ Failure Assessment (SOFA) score at both admission and 48 h. Data were manually extracted from electronic health records and stored in a password-protected database for analysis.

Statistical analysis was conducted using SPSS software (version 26), with continuous variables presented as means and standard deviations and categorical variables as frequencies and percentages. Multivariate logistic regression analysis was performed to identify the independent predictors of TTS, adjusting for age, sex, race, and relevant comorbidities. Odds ratios (OR) and 95% confidence intervals (CI) were calculated for each significant variable. Statistical significance was set at *p* < 0.05.

## 3. Results

A total of 361 patients with sepsis admitted to the medical ICU were included in this study, of whom 24 (6.65%) met the criteria for Takotsubo Syndrome (TTS). The mean age of the cohort was 66.79 years (SD 16.51), with a female predominance, comprising 54.6% of the overall population and 70.8% of the TTS group. Baseline characteristics of the study participants are summarized in Table 1. Patients diagnosed with TTS had a significantly lower mean body mass index (BMI) of 22.72 kg/m^2^ compared with 29.57 kg/m^2^ in patients without TTS (*p* = 0.002). Additionally, hypertension was less prevalent in the TTS group (45.8% vs. 67.0%, *p* = 0.035), while a history of congestive heart failure (CHF) was more common among TTS patients (37.5% vs. 17.0%, *p* = 0.024). A trend toward higher prevalence of smoking in the TTS group compared to the non-TTS group was also observed (37.5% vs. 20.9%, *p* = 0.059). Left Ventricular Ejection Fraction (LVEF) was significantly lower in the TTS group. The etiology of sepsis as identified by positive cultures is presented in Supplementary Table S1.

At ICU admission, patients with TTS exhibited notable hemodynamic differences. The mean shock index, a measure of circulatory status, was significantly higher in the TTS group (1.36 vs. 0.94, *p* < 0.001), indicating a greater degree of cardiovascular compromise. Correspondingly, the mean arterial pressure (MAP) was significantly lower in the TTS group (70.19 mmHg vs. 85.86 mmHg, *p* = 0.001), suggesting reduced perfusion pressures. Peak lactate levels during admission were also markedly elevated in the TTS group (mean 6.76 vs. 4.85 mmol/L, *p* = 0.015), reflecting a state of heightened metabolic stress and hemodynamic instability. Other admission characteristics, such as the Sequential Organ Failure Assessment (SOFA) score, were higher in the TTS group, though this did not reach statistical significance (7.38 vs. 6.05, *p* = 0.095).

Logistic regression analysis identified several independent predictors of TTS among patients with sepsis. Female sex was associated with a significantly higher likelihood of developing TTS (OR 2.718, 95% CI 1.013–7.295, *p* = 0.047), consistent with the known female predominance in TTS. A BMI < 25 Kg/m^2^ was also a significant predictor (OR 0.887, 95% CI 0.812–0.970, *p* = 0.008), suggesting a potential vulnerability in patients with reduced body mass. Additionally, a higher shock index was strongly associated with the development of TTS (OR 3.563, 95% CI 1.134–11.194, *p* = 0.030), underscoring the role of hemodynamic stress in the pathophysiology of TTS. The regression model was statistically significant (χ^2^ = 35.68, *p* < 0.001), explaining 25.2% of the variance in TTS, with a high classification accuracy of 92.7%. Detailed results of the logistic regression analysis are presented in Table 2.

A logistic regression was performed to ascertain the effects of covariates on the likelihood that subjects have Takotsubo Syndrome. The logistic regression model was statistically significant, χ^2^ = 35.68, *p* < 0.001. The model explained 25.2% (Nagelkerke R^2^) of the variance in Takotsubo Syndrome and correctly classified 92.7.0% of cases. Reducing BMI and increasing shock index were associated with a higher likelihood of having Takotsubo Syndrome. Women had a 2.72 times higher likelihood of having Takotsubo Syndrome.

## 4. Discussion

In our study, the patients who developed TTS had a significantly lower baseline BMI. Additionally, lower BMI was independently associated with an increased risk of TTS development. Isogai et al. conducted a retrospective database study involving 14,551 patients with TTS, revealing that in-hospital mortality was highest (9.4%) among those classified as severely underweight (BMI < 16.0 kg/m^2^), followed by those in the mild/moderate underweight category (BMI: 16.0–18.4 kg/m^2^). In contrast, the obese group (BMI ≥ 27.5 kg/m^2^) exhibited the lowest mortality rate [11]. In contrast, a separate retrospective analysis by Desai et al. reported that obese patients hospitalized with TTS had an increased risk of cardiac complications, including myocardial infarction, cardiac arrest, cardiogenic shock, and the need for advanced mechanical circulatory support during hospitalization, although no significant differences in in-hospital mortality were observed between obese and non-obese patients with TTS [10].

Subgroup analysis based on age and BMI (Table 3) confirms the persistence of the obesity paradox in Takotsubo Syndrome among septic patients. In all age groups, patients with a BMI ≥ 25 kg/m^2^ exhibited a lower incidence of TTS compared to those with BMI 25 kg/m^2^, suggesting that obesity remains protective irrespective of age. Notably, the protective effect was observed in both younger (<65 years) and older (>65 years) populations, reinforcing that age does not serve as a confounder in this relationship. A potential explanation for the observed obesity paradox may be a bilinear relationship between BMI and TTS risk, where both extremes of weight—underweight and morbid obesity—may contribute to increased susceptibility to TTS.

While Isogai et al. reported an “obesity paradox” in TTS, in which obese patients had a better prognosis, our study also suggested that obesity may further offer protection against the development of TTS in patients with sepsis [11]. The reason for this protective effect remains unclear, but we hypothesized that it may be influenced by the classification of obesity. Individuals with “central obesity” exhibit heightened SNS activity, whereas those with “subcutaneous obesity” do not, despite being categorized as obese according to BMI [15]. This distinction is crucial because the pathogenesis of TTS is closely linked to increased SNS activity and elevated catecholamine levels. In our patient sample, we speculate that there was a lower proportion of individuals with central obesity compared to those with subcutaneous obesity for the same BMI. Prior studies [16], have shown that patients with central obesity have elevated baseline SNS activity, making them more prone to developing TTS and those with subcutaneous obesity have inherently lower baseline SNS activity which might confer protection from developing TTS.

Our study also reported a higher risk of TTS development in females than males. This finding aligns with other studies, such as those by Deshmukh et al., who reported an odds ratio of 8.8 for the development of TTS in females, especially post-menopausal women, relative to males using the Nationwide Inpatient Sample database [17]. Akashi et al. reported similar findings and hypothesized that men may possess a protective mechanism against the deleterious cardiac effects of catecholamines induced by stress, one of the proposed causes of TTS, in contrast to women [18]. Evolutionary research suggests that men have historically faced greater physical stress than women, potentially leading to the development of various strategies to mitigate the adverse effects of such stressors [19]. Furthermore, the density of adrenergic receptors in the membranes of cardiomyocytes is higher in men than in women. This heightened receptor density may confer enhanced protection against severe catecholamine surges, as the saturation of a significant number of adrenoreceptors occurs more slowly in men than in women (9,10).

## 5. Limitations

Our study had several limitations that warrant consideration. First, the small sample size, particularly among patients with TTS, limits our ability to capture the full spectrum of BMI distributions, particularly at the extremes, potentially underestimating the association. Also, the fact that the data were collected from a single institution may limit the generalizability of our findings and result in underpowered outcomes, making it challenging to detect statistical significance. Second, it must be recognized that the diagnosis of TTS in the ICU is challenging. Several conditions such as post-arrest myocardial dysfunction can present with RWMAs without significant coronary artery disease as typically seen in TTS [20]. The distinguishing factor in this case is normalization of left ventricular function and resolution of RWMAs in subsequent imaging studies. Furthermore, patients in the ICU are often mechanically ventilated and cannot verbalize symptoms of chest pain or dyspnea, hence diagnosis is based on TTE findings and the condition may be underrecognized. Third, our study did not assess the long-term outcomes associated with TTS, which is critical for understanding the complete risk of developing this condition and predictors of long-term mortality in patients with sepsis. Fourth, due to the retrospective nature of our study, data availability (e.g., results of cardiac MRI, frequency of the left ventricular outflow tract obstruction phenotype, and patterns of RWMAs and ballooning) was limited. Because our database only included a single TTE assessment during the ICU stay, it was impossible to determine which patients recovered their RWMAs. Lastly, our analysis does not stratify age groups within sexes concerning the risk of developing TTS or their prognosis, despite previous studies indicating that TTS is most frequently observed in postmenopausal women.

## 6. Conclusions

In this retrospective cohort study, we identified key risk factors associated with the development of TTS in septic ICU patients, including female sex, lower BMI and a higher shock index. Notably, our findings reinforce the presence of an “obesity paradox,” where higher BMI appeared protective against TTS in sepsis, a phenomenon that persisted across age subgroups. This paradoxical relationship may reflect underlying physiological mechanisms such as differences in sympathetic nervous system activity or inflammatory responses, though further investigation is required to elucidate these pathways.

Given the significant clinical implications of TTS in sepsis, early identification of high-risk patients through hemodynamic and metabolic profiling is crucial for optimizing management strategies. Future studies with larger cohorts and mechanistic insights are needed to refine risk stratification and explore potential therapeutic interventions aimed at mitigating the development of TTS in this vulnerable population.

## Figures and Tables

**Table 1:** Population characteristics.

	Entire Cohort N = 361 (100%)	Without-TTS N = 337 (93.35%)	TTS N = 24 (6.65%)	*p* Value
Demographics, Social, and Past Medical History				
Age, mean (SD)	66.79 (16.51)	67.10 (16.37)	62.50 (18.08)	0.285
Female Sex, N (%)	197 (54.6%)	180(53.4%)	17 (70.8%)	0.098 *
BMI, mean (SD)	29.11 (15.85)	29.57(16.27)	22.72 (4.26)	0.002
Hypertension, N (%)	236 (65.6%)	225 (67.0%)	11 (45.8%)	0.035 *
Diabetes, N (%)	148 (41.2%)	141(42.1%)	7 (29.2%)	0.214 *
Hyperlipidemia, N (%)	125 (34.8%)	120(35.8%)	5 (20.8%)	0.137 *
Smokers, N (%)	77 (22.1%)	68 (20.9%)	9 (37.5%)	0.059 *
Cocaine users, N (%)	21 (6.1%)	19(5.9%)	2 (8.3%)	0.648 ^‡^
Alcohol users, N (%)	58 (16.6%)	53(16.3%)	5 (20.8%)	0.570 ^‡^
Cerebrovascular accident, N (%)	81 (22.5%)	76 (22.6%)	5 (20.8%)	0.840 *
Cirrhosis, N (%)	33 (9.2%)	29 (8.6%)	4 (16.7%)	0.258 ^‡^
ESRD, N (%)	29 (8.1%)	28 (8.4%)	1 (4.2%)	0.708 ^‡^
HIV, N (%)	22 (6.2%)	19 (5.7%)	3 (12.5%)	0.176 ^‡^
COPD, N (%)	78 (21.7%)	75 (22.3%)	3 (12.5%)	0.259
CHF, N (%)	66 (18.4%)	57 (17.0%)	9 (37.5%)	0.024 ^‡^
Coronary artery disease, N (%)	62 (17.2%)	56(16.7%)	6 (25.0%)	0.274 ^‡^
Peripheral artery disease, N(%)	26 (7.2%)	24 (7.1%)	2 (8.3%)	0.688 ^‡^
DVT/PE, N (%)	72 (19.9%)	66 (19.6%)	6 (25.0%)	0.596 ^‡^
Atrial fibrillation, N (%)	65 (18.1%)	61 (18.2%)	4 (17.4%)	1.000 ^‡^
**Admission Data**				
MAP at admission, mean (SD)	84.82 (23.19)	85.86 (23.19)	70.19 (17.77)	0.001 +
AKI at admission, N (%)	254 (70.4%)	237 (70.3%)	17 (70.8%)	0.958 *
Peak lactate, mean (SD)	4.99 (4.61)	4.85 (4.56)	6.76 (4.92)	0.015 +
Leukocytes at admission, mean (SD)	14.26 (16.39)	14.33 (16.85)	13.32 (7.76)	0.784 +
Hemoglobin A1c, mean (SD)	6.57(2.44)	6.62 (2.51)	6.11 (1.42)	0.488 +
SOFA at admission, mean (SD)	6.14 (3.699)	6.05 (3.69)	7.38 (3.61)	0.095 +
Hemodialysis required, N (%)	47 (13.2%)	44 (13.2%)	3 (12.5%)	1.000 ^‡^
Developed ARDS, N (%)	20(5.6%)	18 (5.4%)	2 (8.3%)	0.634 ^‡^
Developed PE, N (%)	21 (5.9%)	19 (5.7%)	2 (8.7%)	0.636 ^‡^
Left Ventricular Ejection Fraction (%), mean (SD)	59.88 (14.24)	61.73 (11.89)	34.04 (18.86)	<0.05

^‡^ Fisher’s Exact Test; + T-Test; * Chi-Square Test.

**Table 2:** Logistic regression analysis for Takotsubo Syndrome.

Covariates	Odds Ratios	*p* Value
Age	0.986 (0.959–1.014)	0.321
**Female Sex**	**2.718 (1.013–7.295)**	**0.047**
Hypertension	0.931 (0.321–2.705)	0.896
**BMI**	**0.887 (0.812–0.970)**	**0.008**
Hyperlipidemia	0.756 (0.229–2.493)	0.645
Diabetes	0.692 (0.243–1.970)	0.491
Peak lactate	1.029 (0.939–1.128)	0.542
MAP at admission	0.988 (0.958–1.019)	0.434
**Shock Index**	**3.563 (1.134–11.194)**	**0.030**

**Table 3:** **Subgroup Analysis between Age and BMI** Four subgroups were identified based on BMI and Age, as follows: Group 1: Age < 65 and BMI < 25 (N = 75) Group 2: Age < 65 and BMI ≥ 25 (N = 90) Group 3: Age > 65 and BMI < 25 (N = 88) Group 4: Age > 65 and BMI ≥ 25 (N = 108).

	Group 1	Group 2	Group 3	Group 4
Takotsubo	11/75 (14.67%)	2/90 (2.22%)	7/88 (7.95%)	4/108 (3.70%)
No Takotsubo	64/75 (85.33%)	88/90 (97.78%)	81/88 (92.05%)	104/108 (96.30%)

Chi-Square *p* = 0.0062.

## Data Availability

The data that support the findings of this study are available upon requests from the corresponding authors.

## Supplementary Materials

The following supporting information can be downloaded at: https://www.mdpi.com/article/10.3390/jcm14082635/s1. The etiology of sepsis as identified by positive cultures is presented in Supplementary Table S1.

## Abbreviations

BMI	Body mass index
CHF	Congestive heart failure
CAD	Coronary Artery Disease
DM	Diabetes Mellitus
HR	Hazard ratio
ICU	Intensive Care Unit
LV	Left ventricle
LVEF	Left Ventricular Ejection Fraction
MAP	Mean arterial pressure
OR	Odds Ratios
RWMAs	Regional Wall Motion Abnormalities
RV	Right ventricle
SD	Standard Deviation
SOFA	Sequential Organ Failure Assessment
SNS	Sympathetic nervous system
TTS, TC	Takotsubo Syndrome
TTE	Transthoracic Echocardiograph
