# Supplementary material for: Obesity Paradox in Takotsubo Syndrome Among Septic ICU Patients: A Retrospective Cohort Study
Source: J Clin Med. 2025 Apr 11;14(8):2635. doi: 10.3390/jcm14082635 (PMC12028263; doi:10.3390/jcm14082635)
Supplement: Supplementary file 1 [file jcm-14-02635-s001.zip › jcm-3552108-supplementary.pdf]

Supplement

Supplementary Table 1. Etiology of Sepsis by Culture

Species	Takotsubo Syndrome (N=24)	No Takotsubo Syndrome (N=337)
Bacillus spp.	1	1
Candida spp.	1	3
Citrobacter amalonaticus	1	2
Escherichia coli	2	31
Enterococcus spp.	2	4
Klebsiella pneumoniae	6	18
Pseudomonas aeruginosa	1	8
Staphylococcus aureus	2	37
Streptococcus agalactiae	1	4
Streptococcus mitis	1	2
Streptococcus pyogenes	1	2
Unknown	5	153
Others	-	72
